# The Impact of Emotion Perception and Gaze Sharing on Collaborative Experience and Performance in Multiplayer Games

**DOI:** 10.3390/jemr19020034

**Published:** 2026-03-25

**Authors:** Lu Yin, He Zhang, Renke He

**Affiliations:** 1 School of Design, Hunan University, Changsha 410082, China; yinlu0097@hnu.edu.cn (L.Y.); he_2024hnu@163.com (R.H.); 2 School of Art and Design, Wuhan University of Technology, Wuhan 430070, China

**Keywords:** multiplayer game, collaborative experience, emotion perception, gaze sharing

## Abstract

Compared to traditional offline collaboration, current online collaboration often lacks nonverbal social cues, resulting in lower efficiency and a reduced emotional connection between teammates. To address this issue, this study used a two-player collaborative puzzle game as the experimental setting to explore the impact of two nonverbal social cues, emotion and gaze, on collaborative experience and performance. Specifically, this study designed four collaborative modes: with and without teammates’ facial expressions, and with and without teammates’ gaze points. Sixty-two participants took part in the experiment, and each pair was required to complete these four patterns. Subsequently, we analyzed their collaborative experience through subjective questionnaires, objective facial expressions, and gaze overlap rates. The experimental results revealed that teammates’ gaze could effectively enhance collaborative efficiency, while facial expression is key to optimizing subjective experience. Combining both cues further acquires advantages in cognitive and emotional dimensions, leading to improved performance outcomes. The study also indicated that facial expressions could alleviate the social pressure triggered by shared gaze from teammates. Additionally, the study also examined how personality differences influenced collaborative experiences and performance. The results indicated that individuals with high agreeableness actively seek social cues, leading to more positive collaborative experiences. This study provides empirical evidence for understanding the interactive mechanisms of cognitive and emotional processes during online collaboration, and points the way toward designing adaptive, personalized intelligent collaborative systems.

## 1. Introduction

Online collaboration has become deeply integrated into various domains, including work, education, gaming, and social interactions, enabling multi-person teams to overcome geographical constraints and collaborate efficiently to solve problems. However, while this computer-mediated form of collaboration offers convenience, it also lacks the crucial nonverbal social cues inherent in face-to-face interactions, such as changes in the partner’s facial expressions, body language, and real-time eye contact [1]. The absence of such social cues not only weakens emotional bonds and sense of belonging among team members but also makes it laborious to build a highly efficient shared understanding [2]. The success of collaborative tasks largely depends on users’ comprehension level and reasoning ability about the task, as well as their perception of others’ emotions or intentions [3]. Therefore, reconstructing these missing social cues through existing technology and enabling users to perceive others’ feelings and attention, thereby enhancing the experience and effectiveness of online collaboration, has become a critical issue in human–computer interaction and computer-supported collaborative work.

Current research on such social cues in collaborative settings primarily focuses on two aspects. First, at the emotional level, studies have employed Emojis or virtual avatars to convey feelings of teammates and demonstrated that these elements could effectively foster positive emotions, enhance social presence, and strengthen team cohesion [4,5]. Second, at the cognitive level, existing research typically visualizes teammates’ gaze information to convey attention distribution or attentional states [1,6,7]. Studies indicated that sharing eye gaze significantly enhances the efficiency of target guidance and the coordination of action planning, while simultaneously fostering shared understanding and behavioral engagement among team members [6,7,8]. These studies also suggested that effective collaboration is not merely the simple sum of information or actions but rather depends on the dynamic synchronization of emotional and cognitive states among participants. In addition, as indicated by Aoyama et al. [9], when team members achieve emotional and attentional synchrony, they not only coordinate cognitive resources more effectively but also develop a deeper understanding of collaborative tasks and their interpersonal relationships.

In summary, existing research has confirmed the positive effects of emotion perception and gaze sharing on social activities individually. However, systematic empirical studies remain lacking regarding the specific differences between the two cues and whether their combination influences collaborative experiences and efficacy. Based on this, the present study aims to investigate the mechanisms by which teammates’ emotions and gaze influence experiences and performance. Specifically, the study constructs experimental scenarios through collaborative games and employs a 2 (facial expressions: with and without) × 2 (gaze point: with and without) within-subjects design. It manipulates the activation states of two information channels, facial expressions and eye gaze, to examine their underlying mechanisms influencing collaborative task performance, gaze overlap rates, facial expressions, and user subjective experience. Importantly, individuals may perceive and comprehend these social cues differently based on their personality traits. For instance, research has shown that personality, particularly agreeableness, modulates how people process social information and respond to interpersonal cues [10,11]. Understanding such individual differences is crucial not only for explaining variability in collaborative outcomes but also for informing the design of personalized systems. Therefore, this study further analyzes differences in collaborative experiences across user groups with different levels of agreeableness. The findings obtained in this study aim to provide theoretical and empirical foundations for understanding online collaboration mechanisms and designing efficient collaborative systems.

The primary contributions of this study are threefold. First, for online collaborative games, we innovatively introduced multimodal social cues that encompass teammates’ gaze and facial expression and systematically evaluated how different presentation methods of these cues impact experience and task efficiency. Second, at the methodological level, we constructed a multidimensional experience measurement framework by integrating subjective questionnaire, objective gaze overlap, and facial expressions, providing a methodological reference for future collaborative interaction research. Third, the findings validated that both facial expression and gaze cues play crucial roles in collaborative scenarios, exhibiting an interactive effect that jointly influences team collaboration.

To the best of our knowledge, this is the first attempt to systematically compare two distinct nonverbal cues, facial expression and eye gaze, in collaborative settings. The discovery provides critical empirical evidence and design guidance for developing adaptive, personalized intelligent collaboration systems. The rest of the study is summarized as follows. Section 2 reviews the related work concerning emotion and gaze in collaborative tasks. Section 3 details the experimental settings and evaluation method for the user collaborative experience. Next, Section 4 reports the results under different social cues. Section 5 discusses the results and analyzes the limitations of this study. Finally, Section 6 summarizes the study.

## 2. Related Work

### 2.1. Emotion Perception

Emotion perception refers to the process by which individuals observe others’ external expressions, such as facial expressions and gestures, to perceive and infer their internal emotional states [12]. This complex cognitive process involves not only recognizing external emotional cues but also actively inferring others’ mental states, accompanied by coordinated responses from an individual’s physiological and cognitive systems. Among numerous emotional cues, facial expression is considered one of the most critical nonverbal channels for emotional communication, owing to its immediacy and rich informational content [13]. It not only provides direct evidence for emotion recognition but also serves as highly efficient real-time communication signals based on its dynamic changes. The vast majority of research employs facial expressions as a channel for emotion perception.

Since Ekman et al. [14] proposed the theory of six basic emotions that transcend cultural boundaries, researchers have continuously expanded the frontiers of emotion recognition. Cordaro et al. [15] further discovered that observers can not only identify others’ basic emotions but also accurately discern complex emotional states, such as satisfaction and embarrassment, through facial expressions. However, emotion perception does not occur independently of the environment; the process is often highly dependent on contextual information. Empirical support for this comes from research by Aviezer et al. [16]. Using images of tennis players as experimental material, the study systematically examined the role of context in emotion perception through behavioral data, EEG data, and subjective scales. The results clearly indicated that context is not merely an interfering factor in the perceptual process but an intrinsic and essential component. This foundational research lays crucial groundwork for subsequent understanding of more dynamic and socialized emotion perception scenarios.

As the focus of research shifts toward more interactive and social online game environments, the exploration of the influence of emotion perception on user experience has become an emerging area of interest. Some studies have explored how players utilize existing mechanisms for emotional expression and non-verbal communication in multiplayer games. For example, Ofner [17] systematically cataloged nonverbal communication channels in online multiplayer games, such as preset emotes, gestures, text messages, and appearance customization, revealing how players utilize these tools for emotional expression, strategic communication, and social interaction. Bhide [18] compared two emotional visualization methods, emojis and halos, in multiplayer FPS games, finding that the former could more effectively enhance players’ social presence and empathy. Sekhavat et al. [4] designed a competitive racing game that displayed opponents’ emojis on the interface. The authors combined eye-tracking, facial expression analysis, and subjective reports to measure the impact of visible emojis on player experience and visual attention. Results revealed that when opponents’ emotions were visible, players allocated more attention to emotional cues, which significantly impacted their inner emotions, behavior, and the perception of the challenge and overall satisfaction. It is worth noting that not all attempts to enhance emotional perception significantly improve the experience. For instance, Westerlund [19] added video communication features to a multiplayer online sandbox game and explored the effects on user experience. However, through game experience questionnaires and interviews, the results found that the presence or absence of video mode did not significantly impact the experience.

In summary, existing research has confirmed that emotion perception plays a crucial role in interactive scenarios such as games and has explored the effectiveness of various emotion perception methods. Nevertheless, most current studies remain grounded in relatively static or constrained experimental settings. Systematic and in-depth exploration remains lacking regarding the mechanisms of emotion perception during natural dynamic interactions, particularly in multi-person collaborative scenarios, where one’s own emotions interact with others’ emotions to form shared experiences.

### 2.2. Gaze Sharing

Gaze sharing serves as a core tool for enhancing cognitive and collaborative efficiency in human society. It is not only essential for understanding others’ mental states but also the cornerstone for constructing shared cognitive spaces and achieving efficient interpersonal interactions [20]. With technological advancements, gaze sharing has been widely adopted in collaboration, gaming, and learning domains to enhance the efficiency of remote human–machine interaction by enabling real-time or asynchronous visualization of individual gaze points [21].

Early research focused on exploring visualizations of gaze sharing and validating the fundamental utility in collaborative tasks. For instance, Zhang et al. [22] compared four gaze display methods, including cursor and trail, in a map search task. The study found that gaze sharing significantly enhances collaborative efficiency by helping users rapidly align their attention with their teammates and reducing communication overhead. However, it also highlighted that overly explicit visualizations may cause visual interference and privacy concerns, providing important cautionary insights for subsequent visual presentation design of gaze sharing.

In scenarios involving high-frequency social interactions, such as remote collaboration and online gaming, some studies have examined the complementary role of gaze sharing in nonverbal communication and its impact on social presence. For instance, research by Maurer et al. [1] indicated that in cooperative games, while gaze sharing does not significantly enhance players’ social presence, it qualitatively reduces verbal misunderstandings, increases the clarity of spatial cues, and improves team coordination. Lee et al. [6] compared the effects of gaze sharing versus no gaze sharing in augmented reality videoconferencing, finding that it significantly enhanced mutual understanding of intentions and collaborative rapport. Bai et al. [7] integrated gaze cues with gestures in mixed reality remote guidance, confirming the pivotal role of gaze information in improving target localization efficiency and spatial layout awareness.

Furthermore, studies in more diverse domains, such as driving and education, have also explored the effects of gaze sharing. For example, Trösterer et al. [23] introduced gaze-sharing in driving scenarios. The study revealed that real-time visualization of the front passenger’s gaze points during navigation tasks significantly reduced the driver’s cognitive load and sense of distraction. In learning contexts, researchers employed gaze sharing to facilitate collaborative learning and guide learners’ attention. For example, Hayashi [24] shared teachers’ gaze with students and utilized multimodal behavioral data to analyze learning quality, confirming that gaze sharing enhances collaborative experiences. Moreover, Xu et al. [25] found that asynchronously sharing peers’ gaze hotspots effectively reduces the time online learners spend distracted or confused, thereby enhancing learning performance.

The above research indicates that providing nonverbal gaze cues can effectively reduce team coordination costs, promote understanding of intentions and cognitive synchronization, thereby enhancing task efficiency, communication quality, and social presence across diverse scenarios. However, current research predominantly focuses on the direct impact of gaze sharing on collaborative experiences, without comparing or deeply integrating it with other nonverbal social cues, indicating a lack of systematic exploration.

In summary, the existing literature has separately demonstrated the value of emotion perception and gaze sharing in enhancing online collaborative experiences. However, most emotion perception studies are based on relatively static scenarios, with insufficient research on the mechanisms by which emotions mutually influence each other and affect experiences during collaboration. Meanwhile, gaze sharing research has primarily focused on its efficacy as a single channel, lacking systematic exploration of its integration and comparison with other key social cues (such as emotions). Therefore, whether facial expressions and eye gaze, as two core nonverbal cues, exhibit distinct impacts on collaborative experiences and task performance during collaboration, and whether their combination yields superior collaborative outcomes, remains a critical yet underexplored question. To address this, this study conducted a controlled experiment to systematically investigate the effects of facial expressions and gaze on collaborative experiences and task performance.

## 3. Experiment

### 3.1. Participants

We recruited participants through university bulletin boards, social media platforms (e.g., WeChat Moments, QQ groups), and online gaming communities. The recruitment advertisement stated the purpose of the study, the estimated duration (approximately 40 min), the location (the university’s human–computer interaction laboratory), the compensation (20 RMB), and the basic eligibility criteria (aged 18 years or older, no color blindness or severe visual impairment, basic keyboard operation skills). Interested individuals should complete a brief online screening questionnaire that collects basic information and contact information.

A total of 87 participants expressed interest and submitted the screening questionnaire. To determine the proper sample size, we conducted a power analysis using the G*Power 3.1.9 software, setting a medium effect size (f = 0.25), α = 0.05, and statistical power (1 − β) = 0.9. The suggested total sample size is 30. To guarantee enough power, a total of 87 participants were invited. Based on scheduling availability and pairwise matching, we selected and invited 70 of them (35 pairs) to participate in the experiment. Finally, 66 participants (33 pairs) arrived at the laboratory at the designated time and began the experiment. However, one pair of participants withdrew midway due to personal reasons, and another pair was excluded because one participant consistently covered their face during gameplay, making it difficult to recognize their expressions. Consequently, data obtained from these two pairs were excluded from the analysis, resulting in a final sample of 62 participants (31 females and 31 males; mean age = 25.50 ± 6.86 years). The completion rate among attendees was 93.9% (62/66).

Additionally, prior research has shown that agreeableness is closely linked to team collaboration. Individuals with high agreeableness typically exhibit a stronger perception of social cues [10], deeper emotional responsiveness and a greater willingness to empathize with others [11]. Therefore, this study also incorporates player personality as an influencing factor, aiming to explore differences in collaborative experience among players with varying levels of agreeableness. We employed the subscale of the NEO-FFI inventory [26] to measure each participant’s agreeableness. The NEO-FFI is a well-validated and widely used instrument in personality research, with established reliability and validity. The agreeableness subscale consists of 12 items, with each rated on a 5-point Likert scale (1 = Strongly Disagree to 5 = Strongly Agree). Sample items include: “I tend to trust others,” “I cooperate with others easily,” and “I sympathize with others’ feelings.” Participants completed the scales independently in a quiet laboratory setting before the experiment. The internal consistency of the scale in our sample was good (Cronbach’s α = 0.82). Based on the collected data, participants were divided into two groups according to the median score (Mdn = 3.75): the high-agreeableness group (*n* = 31, score > Mdn) and the low-agreeableness group (*n* = 31, score ≤ Mdn). The independent-samples *t*-test showed a significant difference in agreeableness scores between the two groups (M high = 4.24 ± 0.36, M low = 3.21 ± 0.42, t(60) = 10.31, *p* < 0.001). Section 4.3 will further analyze differences in collaborative experience among participants with varying personality traits.

This study was conducted in accordance with the Declaration of Helsinki and was approved by the Institutional Review Board of the School of Design, Hunan University (No. 2023006). All participants provided written informed consent before the experiment. The consent form explicitly informed participants that their facial expressions and gaze data would be recorded during the experiment for research purposes only and maintained with strict confidentiality. In addition, participants retained the right to withdraw from the study at any time, upon which their data would be promptly deleted from the database.

### 3.2. Experimental Design

The study utilizes the classic two-player cooperative puzzle game “Fireboy and Watergirl” as the base experimental material. The game is a puzzle platformer where two players separately control a Fireboy and a Watergirl on two computers. Each player controls the role by pressing the arrow keys on the keyboard to move it left, right, and jump. Two players must coordinate to collect all gems in the scene and safely reach their respective portals. Based on the original game, we developed a version using Python 3.9.15 and the Pygame library, which handles the graphics rendering and game logic.

The rules of the game are as follows: Both characters can walk on common ground. Watergirl can move safely on the water surfaces, and touching flames will result in death. Conversely, Fireboy can traverse flame zones but cannot come into water surfaces. The green swamp is a lethal zone for both characters and must be avoided by both. Additionally, there are multiple mechanisms requiring cooperation between players. For instance, when operating a lift, players must divide tasks: one activates the switch to create a temporary passage for the other, who must then pass through swiftly and trigger the next mechanism, enabling both to overcome the obstacle together. The primary game objectives are to collect gems matching the operated character’s color (i.e., Watergirl collects blue while Fireboy collects red) and ultimately enter the portal of the same color to complete the game.

Since the primary objective of this experiment is to investigate the impact of two nonverbal cues, facial expressions and eye gaze, on collaborative experience and performance in multiplayer games, we modify the original game by incorporating RemaskNet [27] and GazeRecorder (https://gazerecorder.com/gazerecorder. Accessed on 1 January 2026) for facial expression recognition and eye-tracking. Both techniques utilize ordinary cameras to capture corresponding data. Among them, GazeRecorder is employed to track gaze points, offering high accuracy of less than 1.0 degrees. It stands as one of the most accurate camera-based eye-tracking solutions currently available [28] and has been employed in multiple studies [29,30]. RemaskNet demonstrated superior classification accuracy on several facial expression datasets and has been utilized in numerous studies to analyze emotions [31,32]. The adapted version of the game enables players to see their teammates’ facial expressions or gaze points during gameplay. Since the subsequent analysis will examine the gaze overlap rate and facial expressions of two players during gameplay, these technologies will be employed to collect user data in real-time across all four modes and store it on the computer disk.

**Figure 1:**
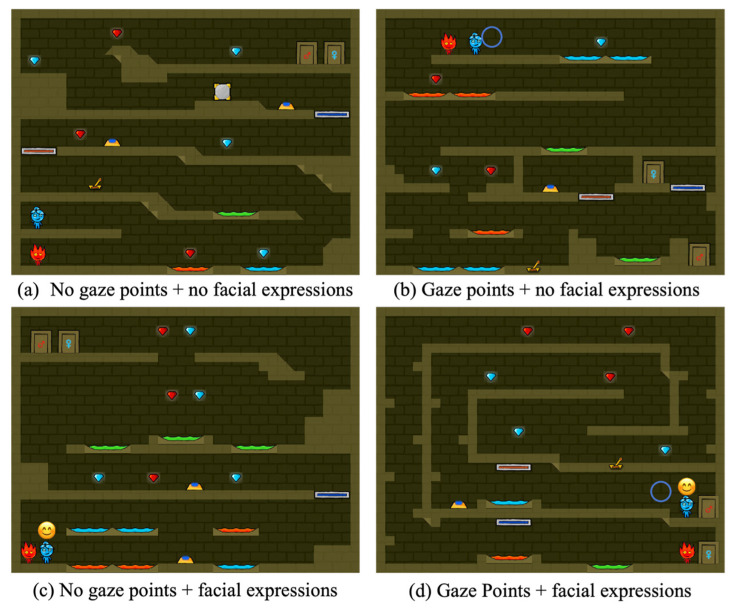
Four game interfaces.

To examine the independent effects and interactions of two nonverbal social cues, this study employed a 2 × 2 within-subjects factorial design (with or without facial expressions and gaze) to construct four experimental conditions: (1) No gaze points and no facial expressions (Figure 1a). The game provided no extra social cues. Players could only interact through the game’s basic interface and voice commands. This condition also served as the baseline for evaluating the effects of other conditions. (2) Gaze points and no facial expressions (Figure 1b). The game tracks and displays teammates’ gaze points in real-time within the interface but does not provide explicit information about facial expressions. (3) No gaze points but with facial expressions (Figure 1c). The game dynamically displayed the partner’s facial expressions as Emoji icons on the character they operated. This condition aims to examine the impact of emotion visualization on the collaborative experience. (4) Gaze points and facial expressions (Figure 1d). The game synchronously displays teammates’ gaze points alongside corresponding emotional Emoji indicators. It aims to establish an integrated nonverbal social environment for exploring the complementary and synergistic effects between facial expressions and gaze information.

To prevent the learning effect from influencing experimental results, we specifically designed four distinct game maps for the four experimental conditions (as shown in Figure 1). To control for potential interference from difficulty differences between maps on dependent variables such as game performance and collaborative experience, we invited six experts in human–computer interaction to test and evaluate each map. Game difficulty was rated using a 7-point Likert scale (1 = “Very Easy,” 7 = “Very Difficult”). Results showed that the mean difficulty ratings for all four maps clustered around 4 (3.83 ± 0.75, 4 ± 0.63, 4.17 ± 0.41, and 3.8 ± 0.41), indicating that each map presented a moderately balanced level of difficulty.

### 3.3. Experimental Procedure

**Figure 2:**
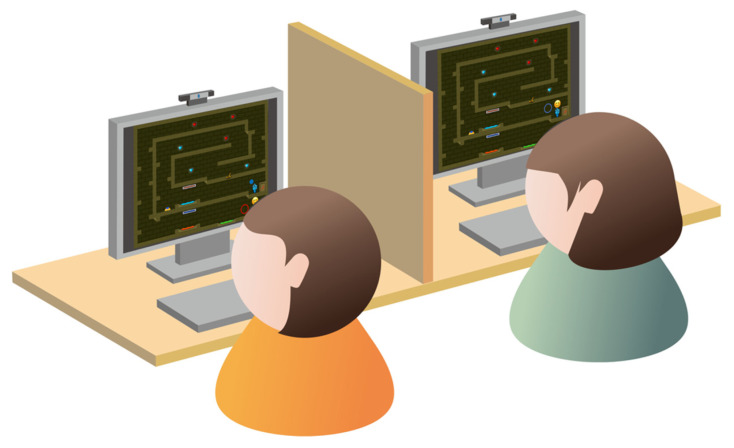
Experimental setting.

Participants engaged in the experiment synchronously in pairs within a laboratory setting, with the specific environmental layout depicted in Figure 2. We choose the pairs of participants primarily based on scheduling availability. During the recruitment phase, interested individuals indicated their available time slots through a screening questionnaire. We initially paired participants based on overlapping availability to ensure both could attend the experiment simultaneously. To control for the potential influence of familiarity on collaborative experience, after forming initial pairs based on the schedule, we further inquired whether the two participants knew each other. If they were acquainted, we reassigned them to different pairs. During the experiment, the two participants sat side by side, with an opaque partition keeping them apart and preventing them from seeing each other’s faces or establishing visual contact. It ensured that facial expressions and gaze information could only be transmitted through the game interface. Each participant operated a computer independently. Two cameras are connected to each computer to separately capture facial expressions and gaze points during gameplay. All computers were interconnected via a local area network, transmitting two types of data in real time: nonverbal cues (including facial expressions and gaze points) and game interaction data (covering the positions of two characters, the status and locations of mechanisms, etc.). Participants were permitted to communicate freely during the experiment to simulate the social context of real online collaboration scenarios. Participants were permitted to communicate freely during the experiment to simulate the social context of real online collaboration scenarios. Based on post-experiment observations of video recordings, verbal exchanges among participants were minimal and predominantly task-oriented and directive in nature (e.g., ‘go left,’ ‘press the red button,’ ‘wait for me’), with little emotional content. The reason might be that participants were unfamiliar with each other before the experiment, and the game task was goal-oriented instead of socially oriented. Furthermore, the frequency of verbal communication appeared to remain consistent across all four conditions, likely due to the uniformity of task structure and difficulty. Therefore, given the minimal verbal communication across all four conditions and the limited emotional information it conveyed, the observed differences in collaborative experience and performance can be primarily attributed to the manipulated non-verbal cues.

The experimental procedure for this study comprised four stages: preparation, training, formal experimentation, and interviews. The specific process was as follows: Upon arrival, participants first signed an informed consent form, then independently completed a basic information questionnaire (covering demographic variables such as gender, personality trait, age, and educational background). The personality trait was obtained by the NEO-FFI scale [26].

Subsequently, researchers directed each participant to conduct the eye-tracking calibration process. Participants first adjusted the seating position to keep their head stable and positioned directly in front of the center of the screen. Then, calibration points appear in order at various locations on the screen, and the participant should focus on each point until it disappears. During this process, the system uses a camera to capture eye images and compute the features, ultimately establishing a mapping model between eye features and calibration point coordinates. Finally, the system can track the participant’s gaze point in real time. The entire calibration procedure typically takes about 1 min. After that, each pair of participants entered the training level together. Researchers systematically explained the game rules, mechanism triggers, and level objectives to both participants simultaneously. Participants may repeat training sessions until they can independently and accurately complete the training level. This phase aims to eliminate the influence of operational proficiency on subsequent experiments. In practice, since the game is relatively simple to operate, all participants reported that they had mastered the basic operations after completing the training level once and did not request additional practice sessions. Therefore, no participants repeated the training level in this study.

Then, the formal experiment commenced, requiring each pair of participants to complete all four game modes. To counteract potential order effects, we presented the game modes in a completely randomized order. Specifically, the system generated a random sequence of the four game modes using the random function in Python, and then players completed them sequentially. Participants were allowed to complete each game mode at their own pace without time restrictions; task completion time was recorded as a dependent variable for subsequent analysis (see Section 4.3). Upon completing each mode, participants immediately filled out the collaborative experience questionnaire. Thereafter, they were required to rest for one minute to alleviate accumulated fatigue before proceeding to the next mode. The average completion time per mode ranged from 86 to 116 s (based on the results in Section 4.3).

**Figure 3:**
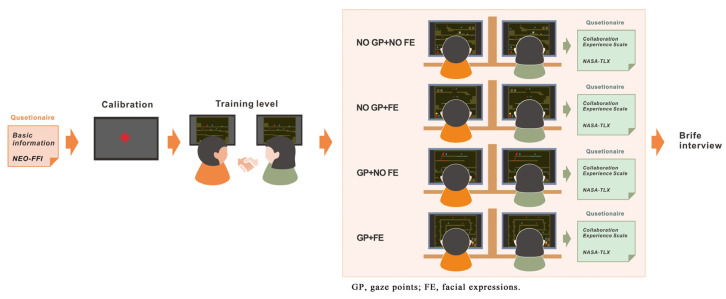
Flowchart of the experiment.

During gameplay, the system simultaneously records participants’ facial expressions and eye-tracking data for subsequent investigation. After completing the four modes, researchers conducted semi-structured interviews with each pair of participants to gain deeper insights into their differing strategies, perceptions, and experiences across different modes. The interviews lasted approximately 5 to 10 min. The purpose was to investigate the participants’ preferences among the four modes, the reasons for their preferences, whether the social cues influenced their communication and coordination, and any challenges or benefits they perceived in each mode. Sample questions included: (1) Which mode did you prefer and why? (2) How did you use your partner’s gaze/emotion information during gameplay? and (3) Did you find the combined information helpful or stressful? All interviews were audio-recorded with participants’ consent and transcribed for qualitative analysis. Figure 3 shows the complete steps of the entire experimental procedure.

### 3.4. Measures

This study combined objective and subjective data to measure collaborative experiences. Objective metrics include task performance, facial expressions, and gaze overlap rates, and subjective measurements encompass collaborative experiences and cognitive load.

#### 3.4.1. Objective Measurements

The collection of objective data aims to simultaneously capture subjects’ natural eye movements and physiological responses during gameplay using non-invasive standard cameras.

First is facial expression. Facial images of participants during gameplay are captured via the front-facing camera, followed by frame-by-frame expression recognition using the Py-feat [31], a feature-rich facial expression analysis tool widely applied across various research domains. The tool outputs intensities for seven basic facial expressions (including happy, surprise, disgust, angry, fear, sad and neutral), serving as objective behavioral indicators of emotional responses. The intensity of each expression ranges from 0 to 1.0. This study adopts the approach of Danner et al. [33] and Zhang et al. [34], who suggested that the peak intensity of each facial expression reflects the user’s most prominent emotional activation level. Therefore, we analyze each expression based on its maximum intensity.

Second is the gaze overlap rate. It was calculated based on the duration of gaze overlap between two subjects at the same fixation region. Specifically, within the two-dimensional screen space, if the distance between participants’ gaze points falls below a set threshold, they are deemed to be looking at the same area. The following formulas (1)–(3) describes the calculation process for the gaze overlap rate.

(1) $$d=\sqrt{{\left(P_{x}-Q_{x}\right)}^{2}+{\left(P_{y}-Q_{y}\right)}^{2}}$$

(2) $$N_{overlap}+=1\;\;if\;d<Thr$$

(3) $$rate=N_{overlap}/N$$

where *P*(*x*,*y*) and *Q*(*x*,*y*) separately indicate the gaze point coordinates of two participants at the same time, *d* is the Euclidean distance between two points, *N_overlap_* denotes the number of gaze overlaps, *Thr* is the threshold, and *N* is the total number of gaze points during gameplay.

Since this experiment uses camera solutions to capture players’ gaze points, which offer lower precision than dedicated eye trackers, we employed a larger threshold than traditional eye-tracking studies, ensuring that instances where participants are looking at the same region are not falsely classified as non-overlapping due to technical limitations. Following the methodology of Schneider et al. [35], we set the threshold to one-quarter of the screen size. In our setup, the screen size was 15.5 inches (diagonal approximately 39.37 cm), so one-quarter of the diagonal corresponds to approximately 9.84 cm. At any given moment, if the distance between the gaze points of two players falls below this threshold, their gazes are deemed to overlap at that instant. This metric can be used to measure joint attention between two subjects, i.e., the ability of multiple individuals to simultaneously focus on the same object or event, which is crucial for effective communication [36].

Third, to objectively measure the synchronization of emotional states between two subjects, we computed the dyad-level correlation of their facial expression intensities. Specifically, we first extracted the intensity vector of the seven basic facial expressions (happy, sad, angry, disgust, fear, surprise, and neutral) for each participant pair at each synchronized time point during gameplay. Then, we calculated the Pearson correlation coefficient between two intensity vectors. The mean value of the correlation coefficients across the duration of each game mode was used as the quantitative metric of emotional synchronization for each pair.

Additionally, we also collect task completion time and number of failures to evaluate game performance across different modes.

#### 3.4.2. Subjective Measurements

This study measures collaborative experience through five dimensions: shared mental models, communication efficiency, emotional resonance, flow, and challenge. Each dimension contains several items, and each was rated on a 7-point Likert scale (1 = Strongly Disagree, 7 = Strongly Agree) (See Appendix A, Table A1 for details).

Shared mental model, as a core concept in team cognitive coordination, was first extended from the proposed by Cannon-Bowers et al. [37] and subsequently refined by Klimoski et al. [38]. This model refers to the organized knowledge structure and mental representations collectively held by team members. It enables members to develop a consistent understanding of team objectives, task requirements, and behavioral expectations, and proactively adjust their own actions to align with others’ needs, thereby achieving highly effective collaboration. The shared mental model scale used in this study was adapted from the Team Mental Model Scale developed by Mathieu et al. [39]. It measures shared understanding of task strategies and role responsibilities (e.g., “My partner and I share a common understanding of how to complete the task”).

Communication efficiency reflects the time and cognitive resources expended by a team in reaching consensus [40]. This study measured communication efficiency using a self-developed questionnaire comprising six items. It assessed subjective perceptions of communication directness, fluency, and effectiveness (e.g., “Our communication is straightforward and free of unnecessary repetition”).

Emotional resonance is a psychological process whereby an individual perceives, understands, and shares others’ emotional states. Its mechanism can be explained through the theory of emotional contagion [41]. This theory posits that individuals achieve emotional convergence by automatically mimicking others’ facial expressions, vocalizations, and behaviors. The Emotional Resonance Scale developed for this study was adapted from the Emotional Contagion Scale [42] and the Interpersonal Reactivity Index (IRI) [43]. It measures the extent to which participants perceive and share their teammates’ emotions (e.g., “I can sense my peers’ emotional shifts during gameplay”).

The flow and challenge dimensions were extracted from the Game Experience Questionnaire (GEQ) [44]. Flow assesses immersion, and challenge measures the perceived difficulty level.

Additionally, whether the introduction of extra nonverbal cues during gameplay significantly increases participants’ cognitive load is a question that warrants exploration. Therefore, we employed the NASA-TLX scale [45] to assess players’ cognitive load across different modes. The scale encompasses several dimensions: Mental Demand, Physical Demand, Temporal Demand, Performance, Effort, and Frustration. Each dimension is scored on a scale ranging from 1 to 20.

## 4. Experimental Results

To investigate the main and interaction effects of teammates’ gaze and facial expressions on collaborative experience, this study employed a 2 (Facial expressions: with vs. without) × 2 (Gaze points: with vs. without) within-factors repeated analysis of variance (ANOVA). Analysis indicators included subjective collaborative experience, cognitive load, facial expressions, task performance, and gaze overlap rate. We report only indicators showing significant differences (*p* < 0.05).

### 4.1. Collaborative Experience

**Figure 4:**
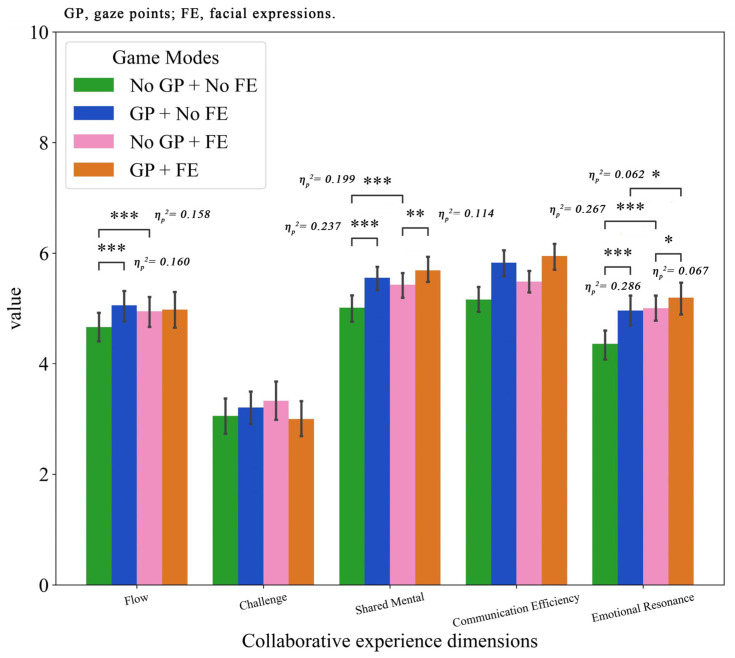
Collaborative experience ratings under different modes. Error bars represent standard deviations. Significant differences are indicated by * *p* < 0.05, ** *p* < 0.01, *** *p* < 0.001.

Figure 4 shows the means and the standard deviations of each dimension of collaborative experience across different game modes.

The results of two-way repeated ANOVA revealed a significant interaction effect between emotion and gaze on the flow dimension (F(1, 61) = 11.835, *p* = 0.001, η_p_^2^ = 0.162). Simple effect analysis further revealed that when gaze points were absent, providing teammates’ facial expressions significantly enhanced flow (MD = 0.286, *p* = 0.001). Conversely, when expression cues were absent, sharing gaze points also significantly enhanced flow (MD = 0.395, *p* = 0.001).

Regarding the challenge dimension, the interaction effect was also significant (F(1, 61) = 7.860, *p* = 0.007, η_p_^2^ = 0.114). However, simple effect analysis revealed no significant difference between the four conditions.

For the shared mental model, the results of two-way repeated ANOVA showed that the main effects of facial expressions (F(1, 61) = 12.790, *p* = 0.001, η_p_^2^ = 0.173) and gaze points (F(1, 61) = 17.227, *p* < 0.001, η_p_^2^ = 0.220) and the interaction effect (F(1, 61) = 7.168, *p* = 0.010, η_p_^2^ = 0.105) were significant. Pairwise comparisons revealed that the shared mental was significantly higher in the emotional condition compared to the No-FE condition (MD = 0.276) and also higher in the gaze condition relative to the no-gaze condition (MD = 0.402). Additionally, simple effect analysis revealed that in the absence of facial expressions, sharing the partner’s gaze significantly enhanced the shared mental (MD = 0.542, *p* < 0.001). When facial expressions were present, the gaze of the partner still provided an additional benefit to a lesser extent (MD = 0.261, *p* = 0.007). Simultaneously, in the absence of gaze cues, facial expressions also enhanced the shared mental (MD = 0.416, *p* < 0.001). These demonstrate that both nonverbal cues form the foundation for constructing shared cognition, with gaze serving as a more efficient tool for cognitive coordination.

Concerning communication efficiency, the results of two-way repeated ANOVA showed that the main effects of facial expressions (F(1, 61) = 7.386, *p* = 0.009, η_p_^2^ = 0.108) and gaze points (F(1, 61) = 33.037, *p* < 0.001, η_p_^2^ = 0.351) were significant. However, the interaction effect was not significant (*p* = 0.157). Pairwise comparisons revealed that the FE condition outperformed the non-FE condition (MD = 0.223), while the GP condition outperformed the non-GP condition (MD = 0.565). These indicate that both cues enhance communication efficiency, with gaze points demonstrating a greater degree of improvement.

In addition, for emotional resonance, the results of two-way repeated ANOVA showed that the main effects of facial expressions (F(1, 61) = 17.055, *p* < 0.001, η_p_^2^ = 0.218) and gaze points (F(1, 61) = 23.460, *p* < 0.001, η_p_^2^ = 0.278) and the interaction effect (F(1, 61) = 8.791, *p* = 0.004, η_p_^2^ = 0.126) were significant. Pairwise comparisons revealed that the FE condition outperformed the non-FE condition (MD = 0.442), while the GP condition outperformed the non-GP condition (MD = 0.397). Additionally, simple effect analysis revealed that FE significantly enhanced emotional resonance regardless of whether gaze cues were present (No GP: MD = 0.648, *p* < 0.001; with GP: MD = 0.235, *p* = 0.049). Furthermore, under conditions with and without facial expressions, the presence of gaze points significantly increased emotional resonance compared to No GP (MD = 0.190, *p* = 0.040; MD = 0.603, *p* < 0.001). The above results suggest that when emotional information from teammates is unavailable, individuals can infer their emotional state based on gaze cues.

### 4.2. Cognitive Load

**Figure 5:**
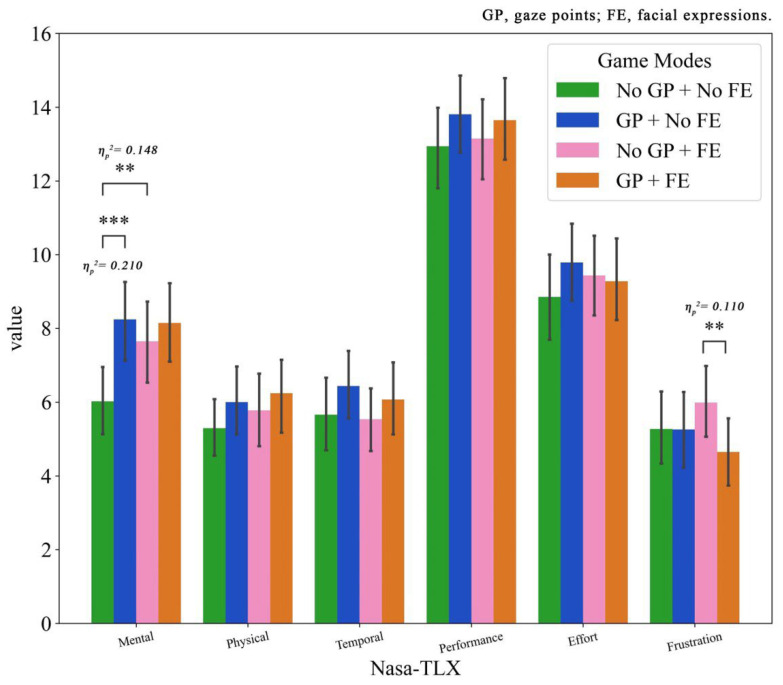
Cognitive load ratings under different modes. Error bars represent standard deviations. Significant differences are indicated by ** *p* < 0.01, *** *p* < 0.001.

Figure 5 shows the means and the standard deviations of each dimension of cognitive load across different game modes.

For mental demand, the results of two-way repeated ANOVA showed that the main effects of facial expressions (F(1, 61) = 5.615, *p* = 0.021, η_p_^2^ = 0.084) and gaze points (F(1, 61) = 9.236, *p* = 0.003, η_p_^2^ = 0.132) and the interaction effect (F(1, 61) = 6.776, *p* = 0.012, η_p_^2^ = 0.100) were significant. Pairwise comparisons revealed that both providing teammates’ emotional and gaze cues led to significantly higher mental demand (MD = 0.766, *p* = 0.021; MD = 1.363, *p* = 0.003). Furthermore, simple effect analysis clarified that in the absence of gaze cues, peer emotional cues still significantly increased mental demanding (MD = 1.629, *p* = 0.002), while in the absence of emotional cues, sharing gaze resulted in an even greater increase in mental demanding (MD = 2.226, *p* < 0.001). These indicate that the introduction of either nonverbal cue alone requires additional cognitive resources, with gaze processing demanding higher psychological needs.

In terms of temporal demand, the results of two-way repeated ANOVA showed that only the main effect of gaze points was significant (F(1, 61) = 4.436, *p* = 0.039, η_p_^2^ = 0.068). Paired comparisons indicated that players perceived stronger time pressure when providing their teammates’ gaze points (MD = 0.653). Furthermore, neither the main effect of emotion nor the interaction effect was significant, indicating that time urgency was primarily associated with the gaze rather than the emotional cues.

In addition, the results of two-way repeated ANOVA showed that only the interaction effect on frustration was significant (F(1, 61) = 5.628, *p* = 0.021, η_p_^2^ = 0.084). Simple effect analysis denoted that when facial expressions were present, peer gaze cues significantly increased frustration (MD = 1.339, *p* = 0.008). However, in the absence of facial expressions, the difference between gaze and no-gaze conditions was not significant. These findings suggest that the combination of both cues may impose additional cognitive or attentional demands, leading to increased frustration when participants are required to process emotional and gaze information simultaneously.

### 4.3. Task Performance and Gaze Overlap Rate

Table 1 shows the means and standard deviations of the number of failures, task completion times, and gaze overlap rates under different game modes. The results of two-way repeated ANOVA showed that only the main effect of gaze points on the number of failures was significant (F(1, 30) = 7.246, *p* = 0.012, η_p_^2^ = 0.195). Pairwise comparisons revealed that the average number of failures was significantly lower under the condition with peer gaze points compared to the condition without (MD = −1.194). These suggest that real-time sharing of visual focus effectively assists two players in anticipating potential risks and coordinating their actions, thereby significantly reducing errors during task execution.

Similarly, gaze points also had a significant main effect on task completion time (F(1, 30) = 10.522, *p* = 0.003, η_p_^2^ = 0.260). Under the condition with gaze points, the average task completion time was significantly shorter than in the condition without (MD = −18.634 s). It directly demonstrates that gaze information is a key factor in enhancing collaborative efficiency.

Furthermore, the analysis revealed a significant main effect of gaze points on gaze overlap rate (F(1, 30) = 9.393, *p* = 0.005, η_p_^2^ = 0.238). Specifically, the average gaze overlap rate was significantly higher under the condition with gaze points compared to the condition without (MD = 0.069).

### 4.4. Facial Expressions

**Figure 6:**
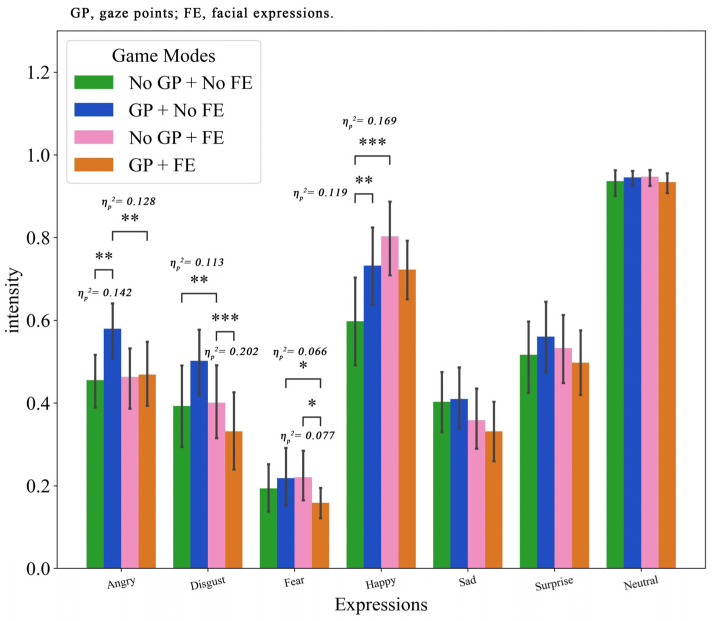
Facial expressions intensities under different modes. Error bars represent standard deviations. Significant differences are indicated by * *p* < 0.05, ** *p* < 0.01, *** *p* < 0.001.

Figure 6 shows the means and the standard deviations of each expression intensity across different game modes. The results of two-way repeated ANOVA revealed that facial expressions and gaze points significantly influenced the intensity of multiple expressions, including angry, disgust, fear, sad, and happy. 

For angry expression, the results of two-way repeated ANOVA showed that the main effects of facial expressions (F(1, 61) = 5.006, *p* = 0.029, η_p_^2^ = 0.076) and gaze points (F(1, 61) = 4.834, *p* = 0.032, η_p_^2^ = 0.073) and the interaction effect (F(1, 61) = 5.274, *p* = 0.025, η_p_^2^ = 0.080) were significant. Pairwise comparisons indicated that the absence of teammates’ emotional cues elicited higher anger expression intensity compared to their presence (MD = 0.052). Similarly, the condition with gaze points triggered significantly greater anger expression than the condition without (MD = 0.065). Simple effect analysis further clarified that emotional cues from teammates effectively mitigate the intensity of angry expressions when simultaneously sharing their gaze points. Specifically, in the presence of gaze cues, the absence of emotional cues significantly increased anger (MD = 0.111, *p* = 0.004). Conversely, in the absence of emotional information, sharing gaze significantly amplified the intensity (MD = 0.124, *p* = 0.002).

In terms of disgust expression, the results of two-way repeated ANOVA showed that the main effect of gaze points (F(1, 61) = 5.567, *p* = 0.022, η_p_^2^ = 0.084) and the interaction effect (F(1, 61) = 8.895, *p* = 0.004, η_p_^2^ = 0.127) were significant. Pairwise comparisons indicated that sharing teammates’ gaze elicited higher disgust expression intensity compared to the no-gaze condition (MD = 0.081). Simple effect analysis further revealed that in the absence of gaze cues, providing emotional cues significantly reduced the intensity of disgust expression relative to the no-FE condition (MD = −0.109, *p* = 0.007). However, when emotional cues were present, gaze points significantly increased the intensity of disgust expression (MD = 0.170, *p* < 0.001). The results implied that when players receive real-time emotional cues from teammates via Emoji, the persistent visibility of their gaze points may cause distraction, leading to negative emotions.

For fear expression, a significant interaction effect was found (F(1, 61) = 4.346, *p* = 0.041, η_p_^2^ = 0.067), with no significant main effects of either facial expressions or gaze points. Simple effect analysis revealed that with gaze cues, the absence of emotional cues significantly increased the intensity of fear expression (MD = 0.059, *p* = 0.042). Moreover, without emotional information, the lack of gaze cues also increased the intensity of fear expressions (MD = 0.061, *p* = 0.028).

Regarding sad expression, only facial expressions showed a main effect (F(1, 61) = 9.528, *p* = 0.003, η_p_^2^ = 0.135). The no-FE condition elicited higher sadness expression intensity than the FE condition (MD = 0.062). Neither the main effect of gaze cues nor the interaction effect was significant, indicating that sad expression primarily relates to the absence of emotional information.

For happy expression, both the main effect of facial expressions (F(1, 61) = 6.161, *p* = 0.016, η_p_^2^ = 0.092) and the interaction effect (F(1, 61) = 9.689, *p* = 0.003, η_p_^2^ = 0.137) were significant. The FE condition elicited higher intensity than the no-FE condition (MD = 0.098). Simple effect analysis revealed that providing emotional information significantly enhanced the intensity of happy expression when peer gaze was absent (MD = 0.205, *p* = 0.001). Similarly, sharing gaze cues alone increased the intensity when emotional information was absent (MD = 0.134, *p* = 0.006).

In addition, Table 2 reports the means and standard deviations of dyad-level correlations of facial expression intensities between partners across the four conditions. The results of two-way repeated ANOVA showed that the main effects of facial expressions (F(1, 30) = 5.224, *p* = 0.030, η_p_^2^ = 0.148) and the interaction effect (F(1, 30) = 6.534, *p* = 0.016, η_p_^2^ = 0.179) on the correlation were significant. Pairwise comparisons indicated that the correlation was significantly lower in the no-FE condition compared to the FE condition (MD = −0.048, *p* = 0.030). Simple effect analysis further clarified that in the absence of peer gaze cues, the no-FE condition showed significantly lower correlation than the FE condition (MD = −0.097, *p* = 0.003). Additionally, when peer facial expressions were absent, the no-GP condition exhibited significantly lower correlation than the GP condition (MD = −0.065, *p* = 0.027).

### 4.5. The Impact of Personality on Collaboration

Table 3 presents the means and standard deviations of collaborative experience, cognitive load, and facial expression intensity for users with different personality traits across various modes. We conducted independent samples *t*-tests to compare the differences between the two groups.

First, we compared the results of subjective collaboration experience and cognitive load among different groups. The results revealed that in the mode without any social cues, individuals with high agreeableness scored higher on mental demand than the low group by approximately 2.23 (t(60) = 2.498, *p* = 0.015). When emotional cues (FE + No GP) were introduced, the value of emotional resonance of those with high agreeableness was higher than that of the low group by about 0.49 (t(60) = 2.073, *p* = 0.042). Similarly, in the mode with gaze points but without facial expressions, the high agreeableness group exhibited around 0.53 higher emotional resonance (t(60) = 2.060, *p* = 0.044). Notably, in the dual-channel mode (FE + GP), the moderating effect of agreeableness was the most extensive and significant. Compared to individuals with low agreeableness, those with high agreeableness significantly scored higher scores across four dimensions: flow (MD = 0.93, t(51.983) = 2.928, *p* = 0.005), perceived challenge (MD = 0.74, t(53.116) = 2.414, *p* = 0.019), emotional resonance (MD = 0.61, t(60) = 2.158, *p* = 0.035), and mental demand (MD = 2.23, t(60) = 2.040, *p* = 0.046).

Furthermore, we compared the results of objective facial expressions. The results showed that participants with high agreeableness exhibited significantly lower intensity in negative facial expressions. Specifically, the intensity of disgust was reduced in both the NO GP + FE mode (t(60) = −2.184, *p* = 0.033) and the GP + NO FE mode (t(60) = −2.113, *p* = 0.039). Additionally, significantly lower disgust expression was also observed in the GP + NO FE mode (t(60) = −2.071, *p* = 0.043) and the GP + FE mode (t(60) = −2.248, *p* = 0.028).

The results indicated that the intensity of negative expressions was significantly lower in the high agreeableness group. In NO GP + FE and GP + NO FE modes, the intensity of disgust expression was lower (t(60) = −2.184, *p* = 0.033; t(60) = −2.113, *p* = 0.039). In GP + NO FE and GP + FE modes, the intensity of angry expression was also significantly lower (t(60) = −2.071, *p* = 0.043; t(60) = −2.248, *p* = 0.028). Conversely, regarding positive expressions, individuals with high agreeableness in the GP + FE condition exhibited significantly higher happy expression intensity than those with low agreeableness (t(60) = 2.037, *p* = 0.046).

## 5. Discussion

This study conducted a 2 × 2 within-subject design to investigate the effects of emotion perception and gaze sharing in the collaborative game. The findings demonstrated that conditions providing social cues improved collaborative experience and task efficiency over the no-cues baseline.

### 5.1. Collaborative Experience and Cognitive Load

The results showed that emotion perception and gaze sharing affected the collaborative experience differently across dimensions. For communication efficiency, both contributed positively, with gaze having a higher effect. It is likely because our task required players to coordinate their spatial positions, and seeing a teammate’s gaze helped them understand intentions and adjust accordingly. However, for shared mental and emotional resonance, a significant interaction effect was observed: these two cues complemented each other. The presence of the other could compensate for the absence of one. For example, when emotional cues were absent, users could infer their partner’s emotional state from the gaze information, thereby enhancing emotional resonance. Conversely, when gaze information was missing, intentions could still be perceived from a partner’s emotions and in-game actions, also serving to improve mutual understanding. It suggests that a teammate’s emotional cues provide a better context for interpreting the intent behind their gaze, and their gaze cues help identify the object or location to which their emotion refers. As one participant stated, “By looking at both where my partner was looking and their expression, I could guess what they wanted.” Furthermore, for Flow, the two cues showed a complementary relationship: the absence of either one significantly reduced players’ immersion. The study also found that emotions played a moderating role in perceived challenge, as they could alleviate the potential pressure induced by gaze sharing. However, some participants reported they felt overwhelmed and thus unable to immerse themselves in the game when two clues existed simultaneously. These support the finding that the dual-channel condition did not significantly surpass single-cue conditions in promoting Flow. It suggests that designers should strike a balance between information richness and interface simplicity. For instance, dynamically adjusting the amount of information displayed based on different task phases. These results confirm the unique contributions of emotion perception to social-emotional resonance and of gaze sharing to cognitive coordination, which aligns with findings from Sekhavat et al. [4] and Lee et al. [6]. More importantly, this study systematically reveals how emotional and gaze cues improve collaborative experience by compensating for each other and working synergistically at both cognitive and affective levels. These provide empirical support for the theoretical framework proposed by Aoyama et al. [9], which posits that the synchronization of emotion and cognition effectively enhances collaboration outcomes.

Regarding cognitive load, the introduction of both emotional and gaze information increased mental demand, with gaze sharing imposing a higher cognitive load. The interaction effect revealed that frustration increased significantly only when both cues were present simultaneously, while gaze sharing alone did not significantly elevate frustration. This suggests that processing two concurrent social cues may impose additional attentional or cognitive demands, potentially leading to information overload or interference. When participants needed to integrate both their partner’s emotional expressions and gaze points, the combined cognitive load may have exceeded their processing capacity, resulting in heightened frustration. According to cognitive load theory [46], individuals have limited working memory capacity and can only process a finite amount of information at any given time. This finding aligns with participant reports that simultaneously attending to both cues felt overwhelming at times. It highlights an important design consideration: while combining multiple social cues can enhance collaboration, designers must strike a balance between information richness and cognitive manageability to avoid overwhelming users.

### 5.2. Task Performance and Gaze Overlap Rate

Concerning task performance, the results indicated that gaze sharing was a key factor in improving collaboration efficiency (reducing failures and shortening completion time). However, the independent contribution and interaction effect of the emotional channel were not significant. These confirm that in tasks requiring spatial and temporal coordination, such as puzzle games, sharing visual focus in real-time is crucial for avoiding misunderstandings and optimizing action paths, which was consistent with the findings of Zhang et al. [22].

However, this does not mean that emotional information is ineffective. First, combining the above results, it is clear that the role of emotion lies not in boosting task efficiency but in optimizing the experience by alleviating the frustration induced by gaze sharing (as shown in the cognitive load results), thereby ensuring the sustainability of collaboration. Second, emotional cues facilitate the understanding of teammates’ intentions. Specifically, gaze cues alone often left players unable to clearly infer their teammates’ intent, whereas the addition of emotional cues made intentions more discernible. For instance, one participant noted: “When my teammate’s character remained still and showed a negative emotion, such as disgust, I could infer that he was unsure how to proceed and needed my help. In contrast, when the character was still but displayed a neutral expression, I understood that he was waiting for me.” These also explain why players paid more attention to their partner’s facial expression icons in FE-only mode, resulting in significantly lower gaze overlap compared to GP modes (with and without FE).

In the dual-channel mode, the gaze overlap rate was nearly identical to the gaze-only condition. Notably, the alignment of gaze in this mode was associated with improved experience. These imply that emotions do not undermine the efficiency value of gaze sharing. Instead, by enhancing players’ understanding of their partner’s intent, team members could maintain high efficiency while achieving better emotional resonance and a stronger shared mental model.

### 5.3. Facial Expression

Facial expression serves as an external indicator of one’s emotions, allowing others to infer their emotional state accordingly. The results of facial expression analysis showed that gaze sharing unaccompanied by emotional cues could lead to negative emotions. In the absence of emotional information, sharing the partner’s gaze significantly heightened emotional responses such as anger, disgust, and fear. It supports the notion that gaze sharing may carry the potential risk of inducing performance pressure or a sense of being monitored [22]. However, the emotional cue played a crucial moderating role. It not only directly increased happiness and reduced sadness, but more importantly, effectively mitigated the negative emotions (e.g., anger) triggered by gaze. These suggest that providing emotional intent interpretation can transform the gaze information from mere spatial coordinates into understandable social signals, thereby reducing the ambiguity and the accompanying negative pressure.

However, the interaction effect between emotion and gaze was not always positive. With the emotion conveyed via an emoji, gaze sharing actually increased feelings of disgust. It indicates that when a teammate’s emotional intent is already clear, the persistent display of their gaze might be redundant or even an interference. On the positive side, emotion and gaze cues demonstrated a complementary effect in enhancing the intensity of happy expression. When one cue was absent, the other could still provide a significant boost, a pattern consistent with their role in promoting the subjective flow experience. In sum, while gaze sharing serves as a cognitive coordinate for collaboration, it can easily generate pressure without an emotional context. This context is crucially provided by emotional cues, which not only boost positive emotions but also help mitigate the potential side effects of cognitive cues. By combining gaze and emotion, we can build a more relaxed, understandable, and positive emotional space for collaboration.

Furthermore, the analysis of dyadic facial expression correlations provides objective evidence of emotional synchronization between partners. As shown in Table 2, the results revealed that facial expression correlations were significantly higher when emotional cues were present. In addition, when emotional cues were absent, the presence of gaze cues significantly increased emotional correlation compared to the no-gaze condition. These findings support the subjective emotional resonance results described in Section 5.1, which implied that gaze information can serve as a compensatory mechanism for inferring the partner’s emotional state. However, the correlation in the dual-channel condition did not surpass the FE-only condition, suggesting that providing peer gaze cues could not further enhance emotional synchronization when emotional cues were already present.

### 5.4. Individual Differences

This study found that the agreeableness personality trait had a moderating effect on the team collaboration. Highly agreeable individuals exhibited deeper emotional engagement, greater task immersion, but also a higher cognitive load when exposed to social cues.

On the subjective level, highly agreeable individuals tended to engage more in social monitoring and reported more pronounced empathic responses. Even in the absence of explicit social cues, they reported higher mental demand, indicating that they spontaneously invest more cognitive resources in inferring and understanding their partner’s emotional state. When provided with clear emotional or gaze cues, they integrated this information more deeply, translating it into stronger emotional resonance, flow, and perceived challenge. This integration was most thorough in the dual-channel condition, continually optimizing their collaborative experience.

Regarding the objective indicators, the facial expressions of the highly agreeable group aligned closely with their subjective reports. Their emotions were less affected by potential social pressure, and thus expressed lower intensity of negative emotions, such as anger and disgust. More importantly, in the information-rich dual-channel mode, they displayed a higher intensity of happy expressions. It demonstrates that social cues are not a burden for highly agreeable individuals but rather satisfy their intrinsic social motivation, making collaboration more positive.

In conclusion, highly agreeable individuals are better at deriving cognitive and emotional benefits from social interaction. This finding highlights the importance of considering personality differences and providing personalized social support in the design of future collaborative systems.

### 5.5. Implications and Limitations

At the theoretical level, the main contribution of this study lies in the systematic revelation and validation of the synergistic mechanism between the emotional and gaze channels. The research confirms that emotional cues provide intent interpretation for gaze information, while gaze information offers spatial grounding for emotional states. The combination jointly promotes a deeper shared mental model and stronger emotional resonance. Furthermore, the study uncovered significant individual differences in social information processing, providing a reference for the design of future collaborative experience prediction models.

From a design practice perspective, this study offers guidance for creating efficient remote collaboration and online social platforms. First, the results suggest that integrating multi-dimensional non-verbal channels during collaboration can yield better synergistic outcomes. Second, the study demonstrates the necessity for developing personalized collaborative systems. Future platforms could tailor the density and presentation of social cues based on user personality or even develop interfaces that dynamically sense user states and adapt accordingly. Finally, design should prioritize providing high-contextual-value social cues rather than simply layering multiple cues. The study confirms that while the dual-channel mode increases cognitive load, it nonetheless yields a net benefit by converting multidimensional information into superior collaborative outcomes and a more positive user experience. Consequently, designers should strike a balance between improving collaborative experience and managing cognitive demands.

This study also has several limitations, which indicate directions for future research. First, the generalizability of the findings is limited by the specific task (a cooperative puzzle game) and sample (predominantly Chinese). Future research should examine whether the complementary benefits of emotion and gaze cues extend to other game genres (e.g., open-world exploration or MOBA games) or non-gaming collaborative scenarios. Furthermore, verbal cues should also be considered, since these scenarios may provoke stronger emotional responses and lead to more frequent verbal exchanges that carry emotional content. Therefore, the impact of verbal cues on collaborative experience also warrants further exploration. Second, while our sample size (*N* = 62) exceeded the minimum required by power analysis to enhance robustness and capture individual differences, it remains relatively homogeneous in terms of cultural background (predominantly Chinese) and task type. Therefore, we suggest that future research recruit even more diverse samples, not merely to increase statistical power, but to ensure generalizability across cultures, age groups, and gaming experiences, and to enable meaningful subgroup analyses. In addition, other personality traits beyond agreeableness also merit investigation. Future studies could incorporate agreeableness as a grouping variable to systematically explore interactions between personality traits and social cues, thereby validating the robustness of our findings. Third, this study captured the eye-tracking data using a webcam solution. Although its effectiveness has been validated in prior research, it may still be inferior to the precision offered by specialized laboratory eye-tracking equipment. Future studies could consider using more professional devices for further verification. Fourth, our LAN-based implementation offers low latency, which may not reflect the challenges of real-world online collaboration. Network latency, bandwidth limitations, and synchronization issues over the internet could affect the effectiveness of sharing various cues. Future research should systematically investigate these effects and explore technical solutions. Fifth, although the four game modes were presented in a random order, the specific sequence for each pair was not recorded, preventing us from analyzing whether starting with a particular mode influenced the results. Future studies could examine potential order effects by recording and analyzing the impact of different starting modes. Finally, extending this research to VR/AR environments represents a promising direction for future work. Previous works have shown that multimodal cues, including gaze, gestures, and facial expressions, could enhance remote collaboration efficiency in VR/AR environments [47,48]. Investigating how emotion and gaze cues interact in immersive settings could inform the design of next-generation collaborative systems.

## 6. Conclusions

This study systematically investigated the influence of two types of non-verbal cues, emotion and gaze, on collaborative experience and performance. The results indicate that they play distinct roles. Specifically, gaze sharing is a core tool for improving collaboration efficiency, whereas emotion is key to optimizing the experience. By providing emotional interpretation, emotions alleviate pressure and enhance emotional resonance and shared mental models. Two-factor analysis further reveals that both cues independently and significantly improve communication efficiency, while exhibiting complementary interaction effects in dimensions such as flow, shared mental model, and emotional resonance. Furthermore, facial expression analysis confirms that emotional cues effectively mitigate the negative emotions triggered by gaze sharing. The study also finds that highly agreeable individuals are more adept at leveraging the dual-channel information, resulting in a better experience.

In summary, this study systematically uncovers the synergistic enhancement mechanism of combined emotional and gaze cues, as well as the associated individual differences. These findings provide a theoretical foundation for the personalized design of collaborative systems.

## Figures and Tables

**Table 1:** Comparison of the number of failures, task completion times, and gaze overlap rates under four modes.

	No FE		FE	
	No GP	GP	No GP	GP
number of failures	4.10 (3.93)	2.84 (3.09)	3.32 (2.83)	2.19 (1.47)
task completion time	116.12 (59.11)	89.25 (49.57)	97.38 (43.16)	86.99 (30.17)
gaze overlap rate	0.362 (0.16)	0.421 (0.14)	0.328 (0.16)	0.408 (0.12)

GP indicates gaze points, and FE is facial expressions.

**Table 2:** Dyad-level correlations of facial expression intensities under four modes.

	No FE		FE	
	No GP	GP	No GP	GP
Correlation	0.522 (0.23)	0.587 (0.23)	0.619 (0.21)	0.586 (0.23)

GP indicates gaze points, and FE is facial expressions.

**Table 3:** Collaborative experience, cognitive load, and facial expressions intensity across different groups with varying levels of interpersonal compatibility under different modes.

Indicators		No GP + No FE		GP + No FE		No GP + FE		GP + FE	
Agreeableness		M High	M Low	M High	M Low	M High	M Low	M High	M Low
Collaborative experience	Flow	4.78 (1.06)	4.54 (1.08)	5.32 (1.02)	4.79 (1.22)	5.10 (0.92)	4.79 (1.21)	5.44 (0.97)	4.52 (1.47)
	Challenge	3.19 (1.35)	2.93 (1.33)	3.49 (1.33)	2.92 (1.07)	3.27 (1.11)	3.40 (1.54)	3.37 (1.41)	2.63 (0.97)
	Shared mental	5.03 (0.89)	4.99 (1.04)	5.69 (0.73)	5.42 (0.94)	5.31 (0.74)	5.55 (1.08)	5.85 (0.90)	5.54 (0.88)
	Communication efficiency	5.12 (1.04)	5.21 (0.88)	5.85 (1.00)	5.80 (0.93)	5.53 (0.80)	5.44 (0.84)	6.04 (0.93)	5.86 (1.01)
	Emotional resonance	4.49 (1.08)	4.23 (1.08)	5.22 (0.96)	4.70 (1.06)	5.25 (0.86)	4.76 (1.00)	5.50 (0.97)	4.89 (1.25)
Cognitive load	Mental	7.13 (3.96)	4.90 (2.98)	9.23 (4.14)	7.26 (4.53)	7.97 (4.22)	7.32 (4.69)	9.26 (4.17)	7.03 (4.42)
	Physical	5.61 (3.20)	4.97 (3.20)	6.48 (3.54)	5.52 (4.07)	6.32 (3.94)	5.23 (3.91)	6.94 (4.11)	5.55 (3.68)
	Temporal	6.35 (4.06)	4.97 (3.35)	6.94 (3.90)	5.94 (3.87)	5.94 (3.56)	5.13 (3.40)	6.81 (4.29)	5.32 (3.68)
	Performance	13.06 (3.92)	12.81 (4.59)	14.03 (4.21)	13.58 (4.38)	14.23 (3.89)	12.06 (4.73)	14.32 (4.08)	12.97 (4.96)
	Effort	9.16 (4.45)	8.55 (5.36)	10.26 (4.11)	9.32 (4.45)	10.45 (3.90)	8.42 (4.93)	10.26 (4.38)	8.29 (4.54)
	Frustration	5.74 (4.06)	4.81 (4.11)	5.42 (4.53)	5.10 (3.83)	6.00 (3.47)	5.97 (4.13)	5.19 (4.34)	4.10 (3.45)
Facial expression	Angry	0.42 (0.27)	0.49 (0.27)	0.51 (0.27)	0.65 (0.25)	0.43 (0.30)	0.49 (0.30)	0.38 (0.31)	0.55 (0.29)
	Disgust	0.33 (0.37)	0.45 (0.41)	0.41 (0.34)	0.59 (0.32)	0.30 (0.32)	0.50 (0.39)	0.29 (0.34)	0.37 (0.40)
	Fear	0.24 (0.24)	0.15 (0.23)	0.24 (0.28)	0.20 (0.25)	0.25 (0.27)	0.19 (0.22)	0.18 (0.16)	0.14 (0.14)
	Happy	0.62 (0.44)	0.58 (0.42)	0.72 (0.37)	0.75 (0.37)	0.82 (0.35)	0.79 (0.36)	0.80 (0.27)	0.65 (0.31)
	Sad	0.41 (0.30)	0.40 (0.31)	0.37 (0.31)	0.45 (0.31)	0.34 (0.30)	0.38 (0.29)	0.32 (0.30)	0.34 (0.28)
	Surprise	0.54 (0.32)	0.49 (0.37)	0.60 (0.31)	0.52 (0.38)	0.54 (0.32)	0.52 (0.34)	0.53 (0.33)	0.47 (0.31)
	Neutral	0.96 (0.04)	0.91 (0.18)	0.96 (0.04)	0.93 (0.09)	0.96 (0.04)	0.93 (0.10)	0.96 (0.05)	0.91 (0.13)

GP indicates gaze points, and FE is facial expressions.

## Data Availability

The data presented in this study are available upon request from the corresponding author. The data are not publicly available due to restrictions, e.g., privacy or ethical.

## Appendix A

**Table A1:** Collaborative experience scale.

Dimensions	Question
Flow	I was completely absorbed in the game (fully immersed).
	2. I lost track of time (lost sense of time).
	3. I was deeply focused on the game.
	4. I felt disconnected from the outside world.
Challenge	5. I found it difficult.
	6. I felt challenged.
	7. I felt time pressure.
	8. I had to put a lot of effort into it.
Shared mental	9. My partner and I shared a common understanding of how to complete the game tasks.
	10. I could accurately predict my partner’s next move during the tasks.
	11. (Reverse-scored) We often had to discuss repeatedly due to different understandings of the goals during the task.
	12. We could always quickly reach a consensus on how to solve difficult parts of the level.
	13. We worked together like a well-coordinated unit when completing tasks.
Communication efficiency	14. Our communication was straightforward, without unnecessary repetition.
	15. I could easily understand my partner’s intentions or instructions.
	16. My partner could also quickly understand what I meant.
	17. (Reverse-scored) We needed to spend a lot of time explaining simple things.
	18. During the tasks, most of our time was focused on progressing rather than correcting misunderstandings.
Emotional resonance	19. I could keenly perceive my partner’s emotional changes during the game.
	20. My partner’s emotional state (e.g., happiness or anger) affected my own emotions.
	21. I felt that our emotions were synchronized at critical moments (e.g., successes or failures).
	22. (Reverse-scored) We mostly completed tasks independently, with little emotional exchange.
	23. This collaboration made me feel emotionally closer to my partner.

## References

[1] Maurer B., Lankes M., Tscheligi M. (2018). Where the eyes meet: Lessons learned from shared gaze-based interactions in cooperative and competitive online games. Entertain. Comput..

[2] Saigot M., Gleasure R., Constantiou I. (2023). Understanding the Affective Layer of Online Collaboration: Toward a Media Affectivity Theory. Academy of Management Proceedings.

[3] Deokar A.V., Meservy T.O., Helquist J., Kruse J. Understanding collaboration success in context of cognitive and social presence. Proceedings of the 2010 43rd Hawaii International Conference on System Sciences.

[4] Sekhavat Y.A., Roohi S., Mohammadi H.S., Yannakakis G.N. (2020). Play with one’s feelings: A study on emotion awareness for player experience. IEEE Trans. Games.

[5] Kim S., Billinghurst M., Lee G., Norman M., Huang W., He J. Sharing emotion by displaying a partner near the gaze point in a telepresence system. Proceedings of the 2019 23rd International Conference in Information Visualization–Part II.

[6] Lee G.A., Kim S., Lee Y., Dey A., Piumsomboon T., Norman M., Billinghurst M. Improving Collaboration in Augmented Video Conference using Mutually Shared Gaze. Proceedings of the 27th International Conference on Artificial Reality and Telexistence and 22nd Eurographics Symposium on Virtual Environments.

[7] Bai H., Sasikumar P., Yang J., Billinghurst M. A user study on mixed reality remote collaboration with eye gaze and hand gesture sharing. Proceedings of the 2020 CHI Conference on Human Factors in Computing Systems.

[8] Delgado D.A., Ruiz J. Evaluation of shared-gaze visualizations for virtual assembly tasks. Proceedings of the 2024 IEEE Conference on Virtual Reality and 3D User Interfaces Abstracts and Workshops (VRW).

[9] Aoyama Lawrence L., Weinberger A. (2022). Being in-sync: A multimodal framework on the emotional and cognitive synchronization of collaborative learners. Front. Educ..

[10] Zhao K., Smillie L.D. (2015). The role of interpersonal traits in social decision making: Exploring sources of behavioral heterogeneity in economic games. Personal. Soc. Psychol. Rev..

[11] Rosa F., Gomes S., Martinho C. Exploring the Impact of Player Personality on Cooperative Game Reward Sharing. Proceedings of the 2024 IEEE Conference on Games (CoG).

[12] Lange J., Heerdink M.W., Van Kleef G.A. (2022). Reading emotions, reading people: Emotion perception and inferences drawn from perceived emotions. Curr. Opin. Psychol..

[13] Ekman P. (1992). An argument for basic emotions. Cogn. Emot..

[14] Ekman P., Sorenson E.R., Friesen W.V. (1969). Pan-cultural elements in facial displays of emotion. Science.

[15] Cordaro D.T., Sun R., Kamble S., Hodder N., Monroy M., Cowen A., Bai Y., Keltner D. (2020). The recognition of 18 facial-bodily expressions across nine cultures. Emotion.

[16] Aviezer H., Ensenberg N., Hassin R.R. (2017). The inherently contextualized nature of facial emotion perception. Curr. Opin. Psychol..

[17] Ofner S. (2021). A Comparative Study of Nonverbal Player-to Player Communication in Online Multiplayer Videogames. Master’s Thesis.

[18] Bhide R.Y. (2023). Exploring Affective Representations in Multiplayer FPS Games and Their Impact on Social Presence. Master’s Thesis.

[19] Westerlund A. (2021). Using Video Communication in Online Multiplayer Games: The Effects of Adding a Video Chat Overlay on the Game Experience in Online Multiplayer Video Games-A Quasi-Experimental Design. Bachelor’s Thesis.

[20] Baron-Cohen S., Tager-Flusberg H., Lombardo M. (2013). Understanding Other Minds: Perspectives From Developmental Social Neuroscience.

[21] Atweh J.A., Riggs S.L. (2025). Gaze sharing, a double-edged sword: Examining the effect of real-time gaze sharing visualizations on team performance and situation awareness. Hum. Factors.

[22] Zhang Y., Pfeuffer K., Chong M.K., Alexander J., Bulling A., Gellersen H. (2017). Look together: Using gaze for assisting co-located collaborative search. Pers. Ubiquitous Comput..

[23] Trösterer S., Gärtner M., Wuchse M., Maurer B., Baumgartner A., Meschtscherjakov A., Tscheligi M. (2015). Four eyes see more than two: Shared gaze in the car. IFIP Conference on Human-Computer Interaction.

[24] Hayashi Y. (2024). Modeling synchronization for detecting collaborative learning process using a pedagogical conversational agent: Investigation using recurrent indicators of gaze, language, and facial expression. Int. J. Artif. Intell. Educ..

[25] Xu S., Hu D., Wang R., Zhang X. PeerEdu: Bootstrapping Online Learning Behaviors via Asynchronous Area of Interest Sharing from Peer Gaze. Proceedings of the 2025 CHI Conference on Human Factors in Computing Systems.

[26] McCrae R.R., Costa P.T. (2004). A contemplated revision of the NEO Five-Factor Inventory. Personal. Individ. Differ..

[27] Pham L., Vu T.H., Tran T.A. Facial expression recognition using residual masking network. Proceedings of the 2020 25th International Conference on Pattern Recognition (ICPR).

[28] Falch L., Lohan K.S. (2024). Webcam-based gaze estimation for computer screen interaction. Front. Robot. AI.

[29] Kelly C.A., Sharot T. (2025). Web-browsing patterns reflect and shape mood and mental health. Nat. Hum. Behav..

[30] Chandrika K.R., Amudha J. (2025). Learner stimulus intent: A framework for eye tracking data collection and feature extraction in computer programming education. Sci. Rep..

[31] Cheong J.H., Jolly E., Xie T., Byrne S., Kenney M., Chang L.J. (2023). Py-feat: Python facial expression analysis toolbox. Affect. Sci..

[32] Wyman A., Zhang Z. (2025). A Tutorial on the Use of Artificial Intelligence Tools for Facial Emotion Recognition in R. Multivar. Behav. Res..

[33] Danner L., Haindl S., Joechl M., Duerrschmid K. (2014). Facial expressions and autonomous nervous system responses elicited by tasting different juices. Food Res. Int..

[34] Zhang H., Yin L., Zhang H. (2024). Using subjective emotion, facial expression, and gaze direction to evaluate user affective experience and predict preference when playing single-player games. Ergonomics.

[35] Schneider B., Sung G. (2024). Is Seeing the Instructor’s Face or Gaze in Online Videos Helpful for Learning?. J. Learn. Anal..

[36] García-Martínez J., Gamboa-Montero J.J., Castro-González Á., Castillo J.C. (2025). Are Robots More Engaging When They Respond to Joint Attention? Findings from a Turn-Taking Game with a Social Robot. Appl. Sci..

[37] Cannon-Bowers J.A., Salas E., Converse S. (1993). Shared mental models in expert team decision making. Individ. Group Decis. Mak. Curr. Issues.

[38] Klimoski R., Mohammed S. (1994). Team mental model: Construct or metaphor?. J. Manag..

[39] Mathieu J.E., Heffner T.S., Goodwin G.F., Salas E., Cannon-Bowers J.A. (2000). The influence of shared mental models on team process and performance. J. Appl. Psychol..

[40] Kraut R.E., Fussell S.R., Brennan S.E., Siegel J. (2002). Understanding Effects of Proximity on Collaboration: Implications for Technologies to. Distributed Work.

[41] Hatfield E., Cacioppo J.T., Rapson R.L. (1993). Emotional contagion. Curr. Dir. Psychol. Sci..

[42] Doherty R.W. (1997). The emotional contagion scale: A measure of individual differences. J. Nonverbal Behav..

[43] Davis M.H. (1983). Measuring individual differences in empathy: Evidence for a multidimensional approach. J. Personal. Soc. Psychol..

[44] Law E.L.C., Brühlmann F., Mekler E.D. Systematic review and validation of the game experience questionnaire (geq)-implications for citation and reporting practice. Proceedings of the 2018 Annual Symposium on Computer-Human Interaction in Play.

[45] Hart S.G., Staveland L.E. (1988). Development of NASA-TLX (Task Load Index): Results of empirical and theoretical research. Advances in Psychology.

[46] Sweller J., Van Merriënboer J.J., Paas F. (2019). Cognitive architecture and instructional design: 20 years later. Educ. Psychol. Rev..

[47] Piumsomboon T., Lee G.A., Hart J.D., Ens B., Lindeman R.W., Thomas B.H., Billinghurst M. Mini-me: An adaptive avatar for mixed reality remote collaboration. Proceedings of the 2018 CHI Conference on Human Factors in Computing Systems.

[48] Teo T., Lawrence L., Lee G.A., Billinghurst M., Adcock M. Mixed reality remote collaboration combining 360 video and 3d reconstruction. Proceedings of the 2019 CHI Conference on Human Factors in Computing Systems.

